# Songbirds as a Potential Vector for Snail Dispersal

**DOI:** 10.1002/ece3.74616

**Published:** 2026-11-04

**Authors:** Theodore J. Zenzal, Kylie Denny, Benjamin Hayden, Samuel Jolly, Kelly Roberts, Lauren Solomon

**Affiliations:** ^1^ U.S. Geological Survey Wetland and Aquatic Research Center Lafayette Louisiana USA; ^2^ School of Biological, Environmental and Earth Sciences University of Southern Mississippi Hattiesburg Mississippi USA

## Abstract

For species with limited mobility, leveraging highly mobile species through animal transport can provide a mechanism for dispersal and range expansion. As such, migratory birds may serve as vectors to disperse mobility‐limited species long distances to potentially novel geographies. To further document the potential use of migratory songbirds as vectors of animal transport for land snails, we provide two accounts of amber snails (*Succinea* sp.) attached to songbirds during post‐breeding migration. Both accounts took place in southwest Louisiana; the first instance occurred on a White‐throated Sparrow ( *Zonotrichia albicollis* ) and the second occurred on a Northern Waterthrush ( *Parkesia noveboracensis* ). Observations like these can improve our understanding of species interactions that may impact ecology, distributions, and life histories.

## Graphical abstract

For species with limited mobility, leveraging highly mobile species through animal transport can provide a mechanism for dispersal and range expansion. As such, migratory birds may serve as vectors to disperse mobility‐limited species long distances to potentially novel geographies. To further document the potential use of migratory songbirds as vectors of animal transport by land snails, we provide two accounts of snails attached to songbirds during post‐breeding migration.

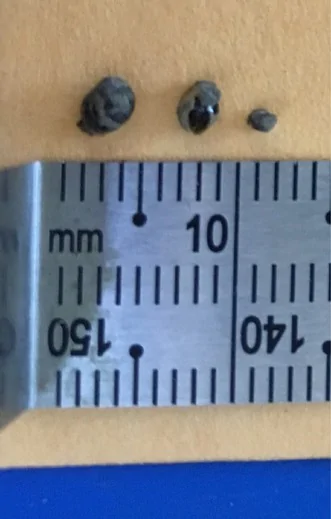

## 1 Introduction

Understanding how animals move is paramount to understanding their ecology, distributions, and life histories (Green and Figuerola 2005; Nathan et al. 2008). Most studies related to movement ecology tend to focus on highly mobile organisms during particular phases of their annual cycle, such as migration or dispersal events. In contrast, few studies have investigated the movement ecology of taxa that tend to have limited mobility. Whereas mobility‐limited species, such as non‐flying invertebrates, plants, fungi, and microorganisms, are unlikely to display large‐scale movements based on their own abilities, these organisms have been known to utilize various methods to move relatively far distances (e.g., Rees 1965; Dörge et al. 1999; Green and Figuerola 2005; Johansson et al. 2021; Yu et al. 2021; Fischer et al. 2025). For example, mobility‐limited animals may use animal transport, wind, water, or other movement assistance methods to disperse or even expand their ranges (e.g., Highsmith 1985; Dörge et al. 1999; Zenzal Jr. et al. 2017; Cho et al. 2018; Yu et al. 2021). However, for some species these instances of long‐distance mobility can be sporadic and not well documented.

Small animals, such as terrestrial, non‐flying invertebrates (e.g., Arachnida, Neodermata, Gastropoda, Bivalvia, Siphonaptera), tend to be mobility‐limited, thereby limiting their ability to disperse, engage in range expansion, or colonize new geographic areas independently. Many invertebrate species have evolved to overcome mobility limitations. For example, spiders take advantage of winds to balloon relatively far distances (e.g., Dean and Sterling 1985; Weyman et al. 2002; Montes and Gleiser 2024). Many parasitic species (e.g., Ixodida, Siphonaptera, Neodermata), as another example, use internal or external animal transport as their primary mechanism of traveling long‐distances at little energetic cost (Mukherjee et al. 2014; Karim et al. 2024; Gładysz et al. 2026). Two common taxa that serve as vehicles for animal transport are mammals, which tend to move across small to moderate spatial scales (i.e., local to regional scales), and birds, which can move across small to large spatial scales (i.e., local to global scales).

Migratory birds travel across relatively large spatial scales twice per year, with movements ranging from hundreds to tens of thousands of kilometers during a single season (Newton 2008). Such long‐distance movements present an opportunity for “hitchhikers” to use migratory birds as a dispersal mechanism to access areas that would otherwise be impossible. For example, Ixodida and Gastropoda species have been found to use birds for animal transport, the latter being transported internally or externally (e.g., Simonová et al. 2016; Zenzal Jr. et al. 2017; Karim et al. 2024). Transport of Gastropoda species has been well documented in waterbirds through internal (e.g., Kawakami et al. 2008; Wada et al. 2012; van Leeuwen et al. 2012; Simonová et al. 2016; Barboza et al. 2022) as well as external means (e.g., McAtee 1914; Roscoe 1955; Simonová et al. 2016; Saito et al. 2023; Green et al. 2023). However, external transport of Gastropoda species by landbirds has been documented less frequently, though evidence suggests that landbirds may serve as a dispersal mechanism for slugs and snails. For example, slugs have been found during autumn migration on the Common Yellowthroat ( *Geothlypis trichas* ) and Northern Saw‐whet Owl ( *Aegolius acadicus* ; Pearce et al. 2012), whereas snails have been found during both migratory and non‐migratory periods attached to the feathers of at least a dozen landbird species (Table 1). Here, we provide two additional instances of snails attached to landbirds during post‐breeding migration in Louisiana, USA.

**TABLE 1:** Landbird species that have been documented with snails attached.

Common name	Scientific name	Source
Bobolink	*Dolichonyx oryzivorus*	Ramsden 1913, 1914
Common Chaffinch	*Fringilla coelebs*	Williamson et al. 1959
Eastern Whip‐poor‐will	*Antrostomus vociferus*	Dundee et al. 1967
Great Tit	*Parus major*	Rusiecki and Rusiecka 2013
Hooded Crow	*Corvus cornix*	Williamson et al. 1959
Indigo Bunting	*Passerina cyanea*	Zenzal Jr. et al. 2017
Lark	Alaudidae	Leege 1898
Meadow Pipit	*Anthus pratensis*	Evans 1915; Lemche 1954^a^
Northern Bobwhite	*Colinus virginianus*	Flock et al. 2018
Northern Waterthrush	*Parkesia noveboracensis*	Williamson et al. 1959
Northern Wheatear	*Oenanthe oenanthe*	Williamson et al. 1959; Harris et al. 1960
Vesper Sparrow	*Pooecetes gramineus*	Huey 1936

^a^ Personal correspondence to Rees (1965).

## 2 Methods and Observations

We captured passerines during post‐breeding migration at a woodlot near Hayes, Louisiana, USA (30.1097° N, 92.9087° W; Figure 1) in 2018 and 2019. The dominant tree species in the woodlot included Chinese Tallow ( *Triadica sebifera* ), Sweet Gum ( *Liquidambar styraciflua* ), and Water Oak ( *Quercus nigra* ). The dominant understory included Poison Ivy ( *Toxicodendron radicans* ), Greenbriers (*Smilax* sp.), Southern Arrowwood ( *Viburnum dentatum* ), Trumpet Creeper ( *Campsis radicans* ), and Grapevine (*Vitis* sp.). We captured birds using up to 17 mist nets (12 m × 2.6 m, 30 mm mesh) placed strategically throughout the study area from 23 August to 29 October. We operated mist nets daily and checked them approximately every 30 min between 0615 and 1320 CDT, unless inclement weather or other extraneous factors prevented the operation of nets. We banded all captured birds with a uniquely numbered metal leg band (U.S. Geological Survey [USGS] issued), then identified the species, age, and sex of each individual as well as collected morphometric measurements (e.g., wing, mass; Pyle 1997). We also assessed the body condition of each individual using a categorical scale for both fat (0–5; Helms and Drury 1960) and muscle (0–3; Bairlein 1995). Finally, we inspected individuals for ectoparasites when time permitted (refer to Karim et al. 2024 for more details). All birds were captured under permission from the USGS Bird Banding Laboratory (24101), the Louisiana Department of Wildlife and Fisheries (LNHP‐18‐020; WDP‐19‐005), and the Institutional Animal Care and Use Committees at the USGS Wetland and Aquatic Research Center (USGS WARC LFT 2019‐05) and the University of Southern Mississippi (17081101).

**FIGURE 1:**
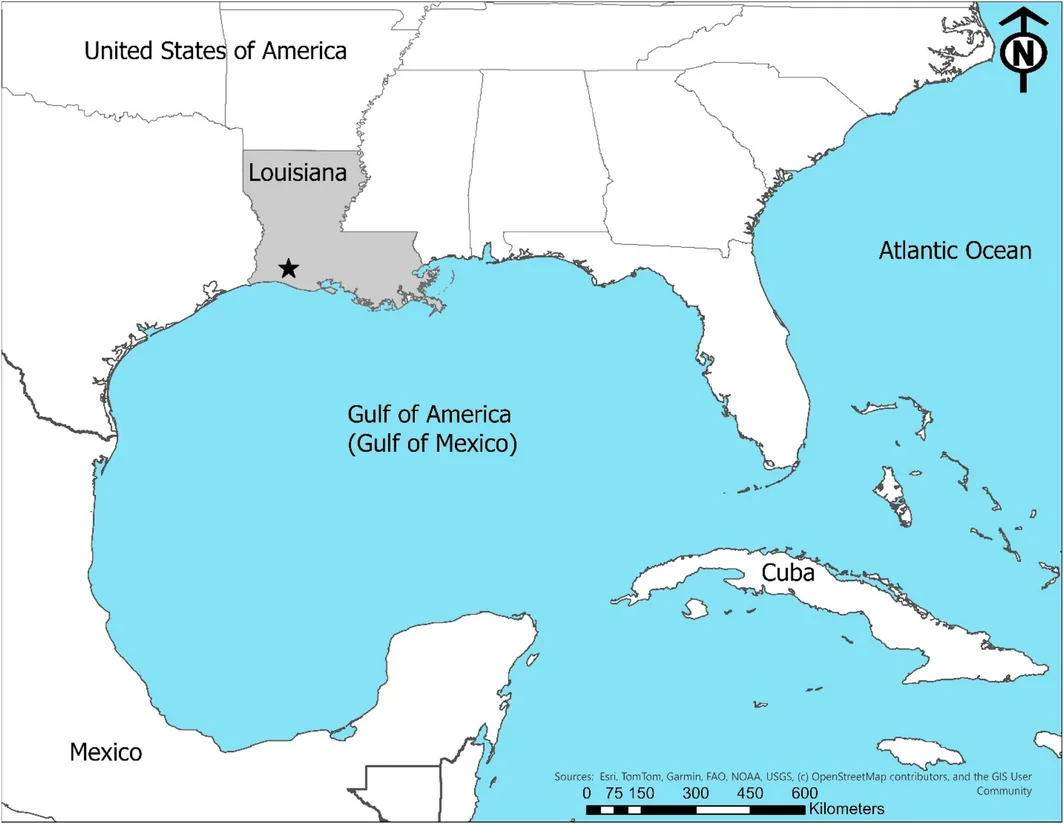
Location of banding station (star) where birds were captured during post‐breeding migration in 2018 and 2019 in southwest Louisiana, USA (gray shading).

During our autumn migration banding sessions in 2018, we performed 1113 ectoparasite inspections on captured birds comprising 30 species and only found one individual with live snails attached. We captured a White‐throated Sparrow ( *Zonotrichia albicollis* ) with four snails attached to the feathers at 0805 CDT on 27 October 2018 during normal banding operations. The sparrow was determined to be aged as a hatch year, but we were unable to determine sex. The individual had a wing chord of 70 mm, a mass of 20.4 g, a fat score of 0 (i.e., no fat observed), and a muscle score of 1 (i.e., little muscle). During an inspection for ectoparasites, LS discovered four snails, which included one relatively large (~10 mm) and three smaller (1–3 mm) individuals, attached to the bird's body contour feathers (Figure 2). The large snail was located on the bird's lower abdomen, and the smaller snails were on the lower back, just above the bird's rump. Based on DNA analysis (LifeScanner; Guelph, Ontario, Canada), two of the snails were identified as belonging to the genus of amber snails (*Succinea* spp.). Additionally, we were able to visually confirm the identity of all four snails belonging to the genus of amber snail (R.T. Dillon, Freshwater Gastropods of North America Project, written communication, 2019). The exact species of the snails were unable to be determined through visual or DNA analysis.

**FIGURE 2:**
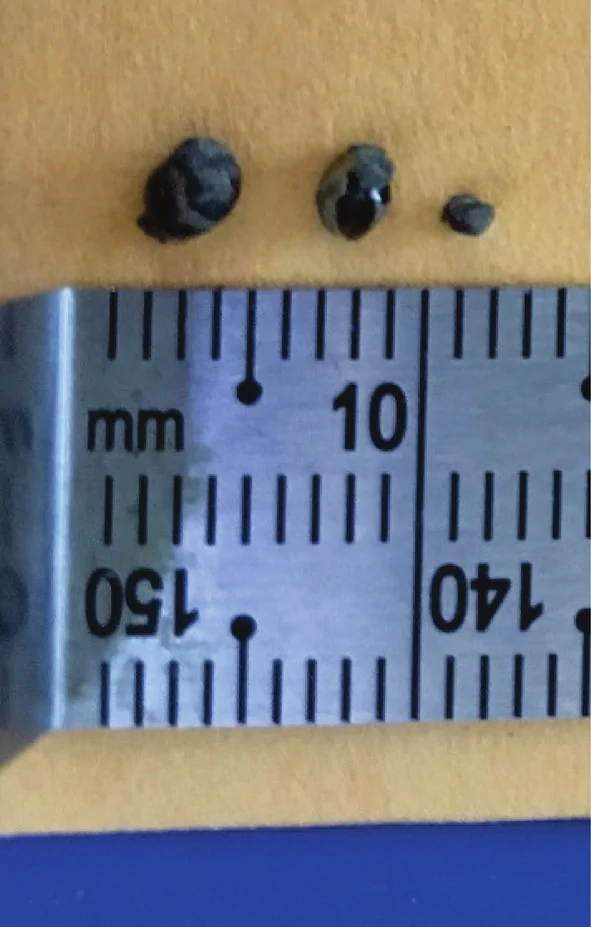
Three snails (*Succinea* spp.) collected from a White‐throated Sparrow captured in southwest, Louisiana, USA during autumn in 2018.

During our autumn migration banding sessions in 2019, we performed 889 ectoparasite inspections on captured birds comprising 51 species and only found one individual harboring a live snail. We captured a Northern Waterthrush ( *Parkesia noveboracensis* ) with one snail attached to the feathers at 0719 CDT on 9 October 2019 during normal banding operations. The waterthrush was determined to be aged as an after‐hatch year, but we were unable to determine sex. The individual had a wing chord of 75 mm, a mass of 19.2 g, a fat score of 3 (i.e., moderate fat), and a muscle score of 1 (i.e., little muscle). During an inspection for ectoparasites, KD found one snail (≤ 3 mm) attached to the ends of the bird's body contour feathers on the center of the back. The snail was not strongly attached to the feathers when removed. Based on DNA analysis (LifeScanner; Guelph, Ontario, Canada), the snail was identified as the Squatty Amber Snail ( *Succinea unicolor* ), a snail known to inhabit Louisiana and other Gulf states (Tryon 1866).

## 3 Discussion

Towards increasing our understanding of snail‐landbird associations, we documented two new instances of snails attached to birds during post‐breeding migration. We provide the first documented evidence of a snail attached to a White‐throated Sparrow, only the second member of the Passerellidae family documented to transport snails (Huey 1936). Additionally, we provide the first record of a snail attached to a Northern Waterthrush in the Americas, and the first record in over 60 years (Williamson et al. 1959; Harris et al. 1960). These new records of snails attached to migratory birds provide more evidence that snails may be using landbirds as a mechanism for dispersal. Ultimately, our observations provide new insights into potential animal transport by invertebrates and add to a growing body of literature documenting these phenomena.

It is unclear when, where, or how the snails attached themselves to the landbirds, but the life history traits of these species may provide some insight. White‐throated Sparrows and Northern Waterthrush are typically found in areas with thick shrubs and can be associated with moist habitats (e.g., swamps, marshes; Falls and Kopachena 2020, Whitaker and Easton 2020). Furthermore, both species are predominantly ground foragers outside of the breeding season, and appear to roost in areas with dense cover (Smith et al. 2008; Falls and Kopachena 2020; Whitaker and Easton 2020). Given these habitats are also suitable for amber snails (Baker 2022), it is possible that the roosting and foraging locations of the sparrow and waterthrush could increase the probability of a snail having the opportunity to attach itself to a landbird.

Snails may use migratory birds as a mechanism of animal transport, which, assuming they survive, can allow for long‐distance dispersal (e.g., Boag 1986; Wesselingh et al. 1999; Saito et al. 2023). In particular, adolescent amber snails may also be able to colonize new areas relatively rapidly given they are known to self‐fertilize (Baker 2022). The two avian species encountered during our banding operations are migratory species captured during post‐breeding migration, although the location of our banding station in southwest Louisiana, USA falls within the non‐breeding range of White‐throated Sparrows. It is possible the individual sparrow with snails attached could have been residing locally in the area, which would provide dispersal at a regional scale—certainly further than the snail could achieve independently (e.g., Lannoo and Bovbjerg 1985; Baur and Baur 1995; Kramarenko 2014). However, this sparrow was never recaptured, suggesting an actively migrating individual rather than a non‐breeding resident. If the sparrow was indeed in migratory disposition, the snails could have traveled as far south as northern Mexico or as far west as Arizona, USA (Falls and Kopachena 2020). In contrast, the Northern Waterthrush does not reside locally and represents a long‐distance migrant stopping over in Louisiana. The nearest stationary non‐breeding area for the waterthrush is located in northern Mexico but could be as far as the northern portion of South America (Whitaker and Easton 2020). Thus, given how far the waterthrush was likely to move after resuming migration, the snail could have been transported to a distant stopover site or possibly even the non‐breeding grounds.

As a result of animal transport, mobility‐limited species can engage in range expansion, which could be especially important given changing climatic conditions that may necessitate exploiting new geographies with more favorable conditions (*sensu* Thomas 2010, Ancillotto et al. 2016). Biological monitoring for snails at bird banding stations may be a proactive approach to better understand the frequency of snails attached to avian hosts. Moreover, the ability to track survival and behavior of attached snails, as done in some studies (e.g., Boag 1986; Flock et al. 2018; Saito et al. 2023), could lead to a better understanding of avian transport as a mechanism for long‐distance snail movements originally proposed by Darwin (1859, 1878).

## Author Contributions

**Theodore J. Zenzal Jr.:** conceptualization (lead), data curation (equal), funding acquisition (lead), investigation (lead), methodology (lead), project administration (lead), resources (lead), supervision (lead), visualization (lead), writing – original draft (lead). **Kylie Denny:** data curation (equal), investigation (supporting), supervision (supporting), writing – original draft (equal). **Benjamin Hayden:** data curation (equal), investigation (supporting), writing – review and editing (equal). **Samuel Jolly:** data curation (equal), investigation (supporting), writing – original draft (equal). **Kelly Roberts:** data curation (equal), investigation (supporting), writing – review and editing (supporting). **Lauren Solomon:** data curation (equal), investigation (supporting), supervision (supporting), writing – review and editing (supporting).

## Funding

This work was supported by the National Oceanic and Atmospheric Administration (NA17NOS4510092) and Alabama Ornithological Society.

## Conflicts of Interest

The authors declare no conflicts of interest.

## Data Availability

All data are included in the manuscript.
